# Characterization of the complete chloroplast genome of *Lonicera similis* (Caprifoliaceae)

**DOI:** 10.1080/23802359.2021.1981161

**Published:** 2021-09-27

**Authors:** Xiao-Mei Wei, Ying Hu, Kun-Hua Wei, Qing-Hua Wu, Yong Huang, Fan Wei

**Affiliations:** a Guangxi Key Laboratory of Medicinal Resources Protection and Genetic Improvement, Guangxi Botanical Garden of Medicinal Plants, Nanning, China; b Guangxi Engineering Research Center of TCM Resource Intelligent Creation, Nanning, China; c College of Pharmacy, Guangxi University of Chinese Medicine, Nanning, China

**Keywords:** *Lonicera similis*, complete chloroplast, phylogenetic analysis

## Abstract

The complete chloroplast genome of *Lonicera similis* Hemsl. has been characterized by reference-based assembly using Illumina paired-end data. The circular complete cp genome is 155,463 bp in length, containing a large single copy (LSC) region of 89,282 bp, a small single copy (SSC) region of 18,661 bp, which are separated by a pair of inverted repeat (IR) regions of 23,760 bp. A total of 129 genes were predicted from the cp genome, including 83 protein-coding genes, 37 tRNA genes, and eight rRNA genes. Phylogenetic analysis reveals that *L. similis* is more closely related to *Lonicera japonica* Thunb. and *Lonicera dasystyla* Rehd. Our result will provide a reference for the phylogenetic relationship, plant identification and resource development and utilization of *Lonicera* species.

*Lonicera similis* is an important traditional Chinese medicine (TCM) plant that belongs to the Caprifoliaceae family. The buds of *L. similis* are used as a substitute for ‘jin yin hua’ (*Lonicera japonica*, a well-known TCM) in the southwest of China and have been used to treat fever, influenza, dysentery in traditional Chinese and Japanese medicine for many years (Xu et al. 1988; Ko et al. 2006). *Lonicera* has abundant variations in morphology and medicinal ingredients. Modern pharmacological studies show that *L. similis* has a higher yield of chlorogenic acid and better antibacterial activity than *L. japonica* (Zhang et al. 2015, 2017), however, the genetic relationship between *Lonicera* species is not clear, e.g. *L. similis* existed as an independent species of the sect. *Nintooa* in the Chinese version flora of China (Xu et al.^1988^). In the updated English version flora of China (Yang et al. 2011), *Lonicera similis* and the other five species were used as synonyms for *L. macrantha* species complex. which seriously hindered the development and application of the *Lonicera* species. Chloroplast genome has been widely used to study the genetic diversity and phylogenetic relationship of plant species in recent years (Jiang 2019), our study will examine the phylogenetic position of *L. similis* in *Lonicera* Genus.

The leaves of *L. similisis* were sampled from Mt. Jingfo, NanchuanCounty, Chongqing City (28.95°N, 107.21°E), Southwest of China. The voucher specimen was deposited at Guangxi Botanical Garden of Medicinal Plants (http://www.gxyyzwy.com/, XM Wei, weixm@gxyyzwy.com, GXMG: CQ00964). Total genomic DNA was extracted according to the modified CTAB method (Doyle and Doyle 1987). Purified genomic DNA was sequenced using the Illumina Hiseq 2500 Platform at Genepioneer Biotechnologies Inc (Nanjing, China). In total, about 24.1 million high-quality clean reads (150 bp paired-end read length) were generated. Quality trimmed were trimmed and assembled into contigs using the program SPAdes v3.10.1 (Bankevich et al. 2012) with the default parameters sets. The annotation was obtained after a BLAST (NCBI BLAST v. 2.2.31) search using the chloroplast genome sequence of *L. japonica* (GenBank accession: MH028738) as a reference sequence. Then for CDS, the annotation results were modified using prodigal v2.6.3 (Hyatt et al. 2010), for rRNA using hmmer v3.1b2 (Potter et al. 2018), and for tRNA using aragorn v1.2.38 (Dean and Bjorn 2004). The finally annotated chloroplast genome of *L. similis* has been deposited into GenBank with the accession number (MW970104).

To reveal the phylogenetic position of *L. similis*, a phylogenetic analysis was performed based on 18 complete chloroplast genomes of *Lonicera* genus. *Heptacodium miconioides* (Genebank accession: NC042739) were chosen as outgroup. These sequences were aligned using the MAFFT v. 7.310 (Katoh and Standley 2013) using the published plastid genomes. The maximum-likelihood (ML) tree was constructed using RAxML v.8.2.10 (Stamatakis 2006) with GTRGAMMA as the nucleotide substitution model and 1000 bootstrap replicates.

The complete assembled chloroplast genome of *L. similis* was 155,463 bp in length. The genome presents a typical quadripartite structure with two IR (each 23,760 bp in length), separated by one SSC and one LSC region (18,661 and 89,282 bp in length, respectively). The overall GC content of *L. similis* plastome was 38.56%. The chloroplast genome encoded a total of 129 genes, including 83 protein-coding genes, 37 transferRNA (tRNA) genes, and eight ribosomal RNA (rRNA) genes. Phylogenetic result (Figure 1) showed that six species of sect. *Nintooa* clustered together, the *L. similisis* was more closely related to *L. japonica* and *L. dasystyla*. However, *L. calcarata* belonged to sect. *Nintooa* was separated from all other *lonicera* species, located in the basal clade, not clustering with other members within the section in taxonomy. In addition, *L. maximowiczii* of sect. *Isika* was firstly clustered with *L. macranthoides*, which belonged to sect. *Nintooa*. The complete chloroplast genome of *L. similisis* contributes to the phylogenetic and evolutionary analysis of *Lonicera* species.

**Figure 1.**
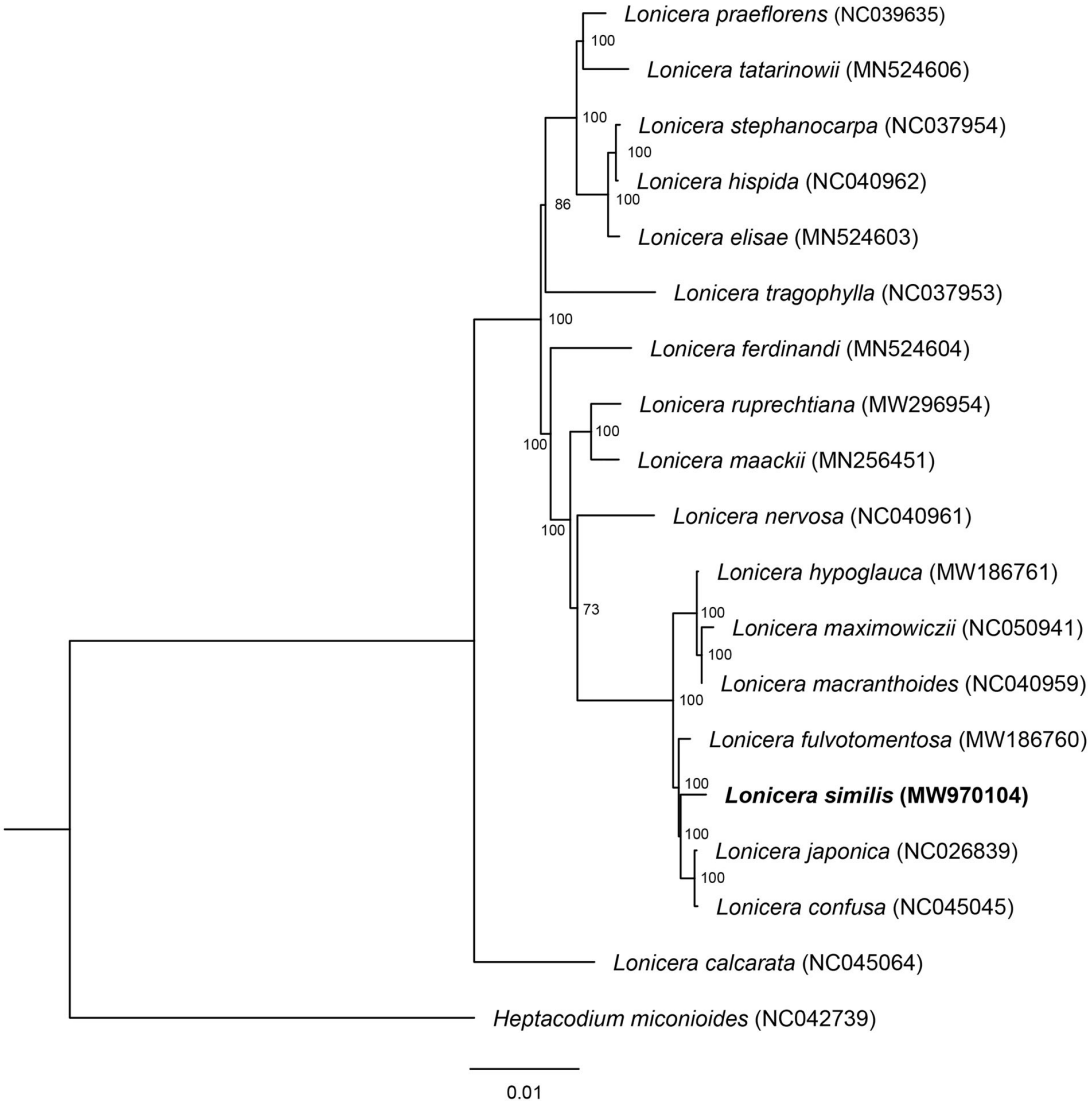
Phylogeny of chloroplast (cp) genome dataset, maximum-likelihood bootstrap support values are shown along the branches.

## Data Availability

The genome sequence data that support the findings of this study are openly available in GenBank of NCBI at (https://www.ncbi.nlm.nih.gov/nuccore/MW970104) under the accession No. MW970104. The associated BioProject, SRA, and Bio-Sample numbers are PRJNA738588, SRR14860699, and SAMN19734401 respectively.
